# Microwave‐assisted extraction optimization of sesquiterpene lactones from *Inula helenium* roots: A sustainable approach to reduce energy consumption and carbon footprint

**DOI:** 10.1002/fsn3.3775

**Published:** 2023-10-13

**Authors:** Fahriye Şeyma Özcan, Hilal Dikmen, Nihat Özcan, Özlem Çetin, Mustafa Çelik, Antoaneta Trendafilova

**Affiliations:** ^1^ TUBITAK Marmara Research Centre Life Sciences Kocaeli Turkey; ^2^ Department of Food Engineering, Faculty of Chemical and Metallurgical Engineering Yildiz Technical University Istanbul Turkey; ^3^ Department of Biotechnology, Faculty of Science Selcuk University Konya Turkey; ^4^ Advanced Technology Research and Application Center Selcuk University Konya Turkey; ^5^ Institute of Organic Chemistry with Centre of Phytochemistry Bulgarian Academy of Sciences Sofia Bulgaria

**Keywords:** antimicrobial, carbon emissions, microwave‐assisted extraction, optimization, SEM, sesquiterpene lactones

## Abstract

*Inula helenium* roots are consumed as natural flavor components and raw or cooked as food, and their extracts are rich in sesquiterpene lactones such as alantolactone (AL) and isoalantolactone (IAL), which have recently attracted great attention due to their pharmacological properties. The industrial utilization of these compounds requires the development of green, efficient, cost‐effective, and sustainable extraction protocols. Therefore, this study focused on the optimization of microwave‐assisted extraction (MAE) process variables using Face‐Centered Central Composite Design (FC‐CCD). Then, maceration was applied as a conventional technique, and these techniques were compared in terms of extraction efficiency, morphological changes, antimicrobial activities, carbon emissions, and energy consumption. As a result, optimal MAE conditions, i.e., EtOH: water ratio (*X*_1_) = 100:0, liquid/sample ratio (*X*_2_) = 30:1 mL/g, microwave power (*X*_3_) = 300 W, and irradiation time (*X*_4_) = 5 min, were obtained with AL and IAL yields of 54.99 ± 0.11 (mg/g) and 48.40 ± 0.19 (mg/g), respectively. The extract obtained by MAE had similar or better activity than positive controls in most cases and formed the largest inhibition zones against *E. coli* (29.5 ± 0.71 mm) and *A. niger* (34.75 ± 1.06 mm). Morphological changes of *I. helenium* roots after extraction were observed by scanning electron microscopy. Additionally, MAE was 43.4 times faster than maceration, resulting in 228.6 times less energy consumption and carbon emissions. Based on these findings, it is recommended to use MAE as an industrial green technique for the extraction of sesquiterpene lactones with potential applications in nutraceuticals and food products in terms of sustainable economy and environmental protection.

## Graphical abstract

The objectives of this study were to investigate the effects of microwave‐assisted extraction (MAE) process parameters (temperature, time, S:S ratio, power, and ethanol concentration) on the sesquiterpene lactone recoveries using single‐factor experiments; optimize the MAE conditions using face‐centered composite design; identify alantolactone and isoalantolactone in the extract at optimal conditions using liquid chromatography–tandem mass spectrometry (LC–MS/MS); evaluate the in vitro antibacterial and antifungal activities of Inula helenium root extract; determine energy consumptions and carbon emissions; compare with a conventional technique (maceration); and confirm by SEM images.

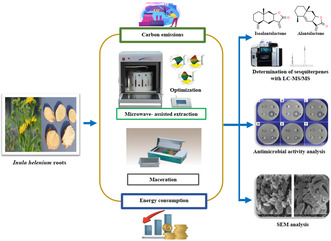

## 1 INTRODUCTION

The genus *Inula* L. consists of 78–100 species found in Europe, Asia, and Africa and is known for the great therapeutic potential of its phytochemical compounds (Buza et al., 2022). Furthermore, *I. helenium* L., a herbaceous perennial medicinal plant belonging to the genus *Inula* L., is rich in eudesmane‐type sesquiterpene lactones, mainly alantolactone (AL) and isoalantolactone (IAL), which have recently attracted attention due to their pharmacological properties, including hepatoprotective, anti‐inflammatory, antitumor, antibacterial, antidematophytic, antifungal, and anticancer activities (Chi et al., 2016; Trendafilova et al., 2010). Moreover, the Food and Drug Administration (FDA) allows the use of alcoholic beverages made from *I. helenium* roots and rhizomes as natural adjuvants and flavoring components in foods, and the Council of Europe (CE) defines *I. helenium* as a natural food flavoring (Food and Drug Administration, 2022). In addition, flowers, leaves, and roots of *Inula* species are consumed as food raw or cooked in Turkey (Ertuğ, 2014).

Considering their great importance, selecting the potentially most efficient extraction technique for certain bioactive compounds is always a challenging task. Recently, there has been a need to develop new environmentally friendly advanced technologies to use them safely, reduce their impact on the environment, and maximize the extraction efficiency of bioactive compounds. These green extraction technologies should aim to increase the yield of extraction with shorter operating times for the isolation of bioactive compounds as well as lower energy consumption, contributing to improve environmental performance (Chemat et al., 2017). Based on all these factors, microwave‐assisted extraction (MAE) has been demonstrated to have great potential for application in the food industry as compared with conventional techniques (Milić et al., 2022).

Although emerging techniques are faster and more efficient compared with conventional methods, the implementation cost of MAE is relatively low compared with that of other techniques. Additionally, recovery yields using MAE are mostly affected by factors such as temperature, the liquid/sample (L:S) ratio, irradiation power, irradiation time, and solvent concentration. Therefore, it is mandatory to optimize the extraction factors to obtain optimum yields. Response surface methodology (RSM), a statistical and mathematical technique that uses a set of experimental data, is often used to develop an adequate functional relationship between the process parameters and responses (Alara et al., 2018). In this study, the face‐centered central composite design (FC‐CCD) was used to create the experimental design matrix. In this study, the FC‐CCD was chosen as the method to optimize sesquiterpene lactones for some reasons. Primarily, the experimental design optimization approach is often considered to reduce costs and time for developing a pharmaceutical formulation with several variables. The Lagrangian approach, simplex method, and RSM are used for optimizing the formulation. However, among these methods, the second‐order response surface methodology, which includes the central composite design, is frequently used because it can handle many independent variables simultaneously. Moreover, the FC‐CCD, which is constructed from a full or fractional factorial design with star points, provides for better estimation of terms of an order greater than one (Tung et al., 2014).

To the best of our knowledge, no research has been reported until now on the optimization of MAE process parameters by using RSM, especially for this plant species. Furthermore, there are no reported data in the scientific literature on a comparative analysis of conventional (maceration) and green extraction techniques (MAE) applied with the intention of isolating the sesquiterpene lactones from *I. helenium* L. Therefore, the objectives of this study were to investigate the effects of MAE parameters (temperature, time, L:S ratio, power, and ethanol concentration) on the sesquiterpene lactone recoveries using single‐factor experiments; optimize the MAE conditions using FC‐CCD; identify AL and IAL in the extract at optimal conditions using liquid chromatography–tandem mass spectrometry (LC–MS/MS); evaluate the in vitro antibacterial and antifungal activities of IHRE; compare with a conventional technique (maceration); and confirm with scanning electron microscope (SEM) images.

## 2 MATERIALS AND METHODS

### 2.1 Raw materials and chemicals

*I. helenium* L. roots were collected from Kırşehir Province in Turkey (39°18′26″N, 34°18′25″E, Date: 31/07/2022), dried naturally at room temperature, finely ground, and passed through a 0.15‐mm stainless steel sieve. The moisture content of *I. helenium* was 9.72% (AOAC, 2000).

Acetonitrile, ethanol, methanol, and formic acid were purchased from Merck (Germany). Distilled water for preparing all the required solutions was from a Milli‐Q ultrapure water purification system (Millipore, France).

### 2.2 Microwave‐assisted extraction

#### 2.2.1 Single‐factor experiments

**FIGURE 1:**
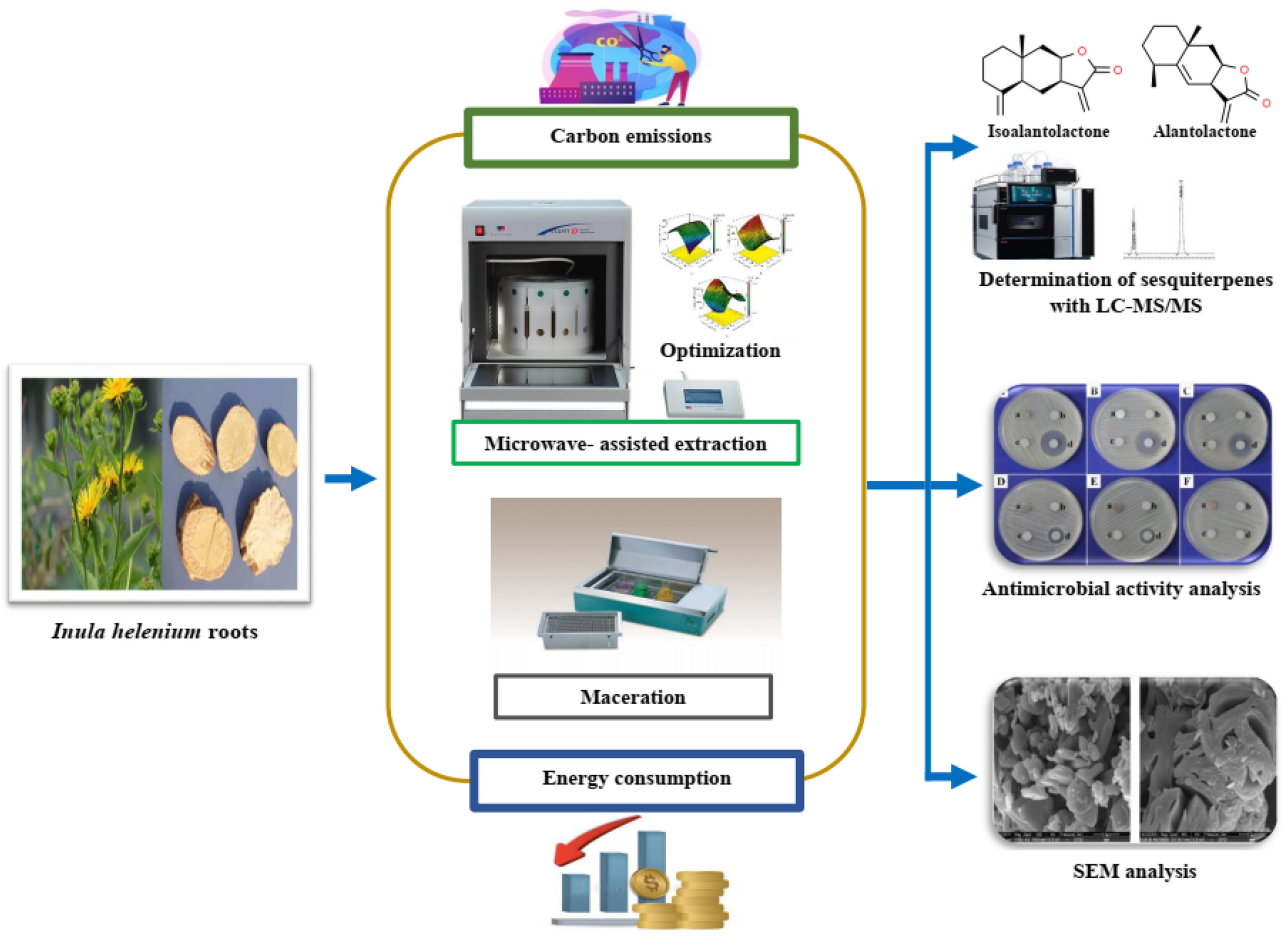
Schematic representation of the experimental setup.

Single‐factor experiments were conducted to assess the influences of microwave power (100, 200, 300, 400, and 500 W), time (1, 3, 5, 7, and 9 min), ethanol/water ratio (0:100, 25:75, 50:50, 75:25, and 100:0), and liquid/solid (L:S) ratio (10:1, 20:1, 30:1, 40:1, and 50:1 mL/g) on the extraction yields of the AL and IAL contents of *I. helenium* L. roots. Microwave‐assisted extraction was performed in a microwave digestion system (Milestone Start D, Sorisole, Bergamo, Italy). All the experiments were carried out in triplicate. A schematic diagram of the whole experimental setup is shown in Figure 1.

#### 2.2.2 Experimental design and optimization

The FC‐CCD was employed in this study to optimize the process parameters and clarify both individual and interactive influence parameters on the extraction yield of AL and IAL from *I. helenium* L. roots.

The experimental design was prepared using Design Expert Version 7 software (State‐Ease Inc.). In this design, a four‐factor, five‐level FC‐CCD with six replicates at the centre point consisting of 30 experiments was performed to enhance the MAE conditions (Table 1). The studied independent variables were *X*_1_ (ethanol/water ratio, v/v), *X*_2_ (solvent/sample ratio, v/w), *X*_3_ (irradiation power, watt), and *X*_4_ (irradiation time, min). The monitored responses (dependent variables) were *Y*_1_ (AL; mg/g) and *Y*_2_ (IAL; mg/g). Desirability indices were created to obtain the optimum experimental conditions to maximize the extraction of sesquiterpene lactones from *I. helenium* roots. Validation experiments were operated in three different runs using parameters suggested by the experimental design, and the AL and IAL contents of *I. helenium* root extracts prepared under optimized conditions were compared with those predicted by the regression model.

**TABLE 1:** Experimental conditions and results of AL (Y1) and IAL (Y2) obtained by MAE of *Inula helenium L*. roots.

Run	*X*_1_ (A): Ethanol/water	*X*_2_ (B): Solvent/sample (mL/g)	*X*_3_ (C): Power (watt)	*X*_4_ (D): Time (min)	Response 1 AL (mg/g)	Response 2 IAL (mg/g)
1	100.00	30.000	300.00	5	54.99 ± 0.11	48.40 ± 0.19
2	50.000	30.000	300.00	5	28.14 ± 0.02	19.87 ± 0.24
3	25.000	40.000	200.00	7	6.70 ± 0.03	3.58 ± 0.25
4	25.000	20.000	200.00	3	2.91 ± 0.03	1.82 ± 0.18
5	50.000	30.000	100.00	5	25.34 ± 0.11	12.76 ± 0.08
6	75.000	40.000	200.00	7	32.90 ± 1.61	28.57 ± 0.19
7	75.000	20.000	400.00	3	29.65 ± 0.73	24.21 ± 0.44
8	25.000	20.000	400.00	3	2.87 ± 0.07	1.95 ± 0.83
9	75.000	20.000	400.00	7	35.88 ± 0.14	30.11 ± 0.04
10	50.000	30.000	300.00	1	30.05 ± 0.47	18.34 ± 0.59
11	50.000	30.000	500.00	5	32.31 ± 1.14	20.09 ± 0.82
12	50.000	30.000	300.00	5	28.76 ± 0.12	20.24 ± 0.72
13	50.000	10.000	300.00	5	15.05 ± 0.16	8.71 ± 0.08
14	50.000	30.000	300.00	9	26.42 ± 0.12	24.18 ± 0.98
15	75.000	40.000	400.00	3	36.11 ± 0.02	31.84 ± 0.99
16	25.000	40.000	400.00	3	13.23 ± 0.31	6.86 ± 0.2
17	50.000	30.000	300.00	5	33.24 ± 0.58	22.40 ± 0.38
18	25.000	20.000	200.00	7	4.04 ± 0.08	2.40 ± 0.05
19	75.000	40.000	400.00	7	36.09 ± 0.52	31.19 ± 0.06
20	75.000	40.000	200.00	3	39.71 ± 1.09	33.95 ± 0.23
21	50.000	50.000	300.00	5	17.35 ± 0.16	14.11 ± 0.39
22	50.000	30.000	300.00	5	34.44 ± 1.85	24.37 ± 1.26
23	25.000	20.000	400.00	7	20.44 ± 0.31	11.26 ± 0.47
24	0.0000	30.000	300.00	5	5.00 ± 0.05	3.83 ± 0.03
25	25.000	40.000	200.00	3	17.74 ± 0.04	10.51 ± 0.31
26	25.000	40.000	400.00	7	29.55 ± 1.30	16.52 ± 0.00
27	75.000	20.000	200.00	7	32.41 ± 0.11	28.67 ± 0.86
28	50.000	30.000	300.00	5	27.62 ± 0.07	19.08 ± 1.37
29	75.000	20.000	200.00	3	35.85 ± 0.02	29.98 ± 0.59
30	50.000	30.000	300.00	5	27.46 ± 0.55	17.63 ± 0.18

A second‐order quadratic equation model was suggested to elucidate the responses as a function of the independent factors, as given below (Tekgül & Baysal, 2019):

(1) $$Y=\beta_{0}+\sum_{i=1}^{n}\beta_{i}X_{i}\sum_{i=1}^{n}\beta_{\mathrm{ii}}X_{i^{2}}+\sum_{i=1}^{n-1}\sum_{j=i+1}^{n}\beta_{\mathrm{ij}}X_{i}X_{j}$$

where *Y* is the response variable, *Y*_*1*_ denotes AL content (mg/g), *Y*_*2*_ refers to IAL content (mg/g), and *Xi* represents the coded independent variables (*X*_1_ = ethanol/water ratio, *X*_2_ = L:S ratio, *X*_3_ = irradiation power, and *X*_4_ = irradiation time). *β*_0_ is the value of the constant coefficient, whereas *β*_i_, *β*_*ii*_, and *β*_ij_ are the linear, quadratic and interactive regression coefficients, respectively. The *k*‐value is the number of independent variables. The test of statistical significance was performed at a 95% confidence level.

### 2.3 Maceration

Conventional extraction of the sesquiterpene lactones was conducted on 1 g of dry plant material mixed with 99% ethanol with an L:S ratio of 30:1 (v:wt) as an optimized ratio resulting from MAE trials. Extraction was performed in a water bath (Nüve, ST 402, Turkey) to control temperature. Experiments were conducted in triplicate at 25°C at different times (days 1, 2, 3, 4, 5, 6, and 7), then filtered before being submitted for LC–MS/MS analysis. Maceration was performed with some modifications in the liquid/solid ratio and extraction time, according to Fernández‐Ronco et al. (2013).

### 2.4 Determination of sesquiterpene lactones

**FIGURE 2:**
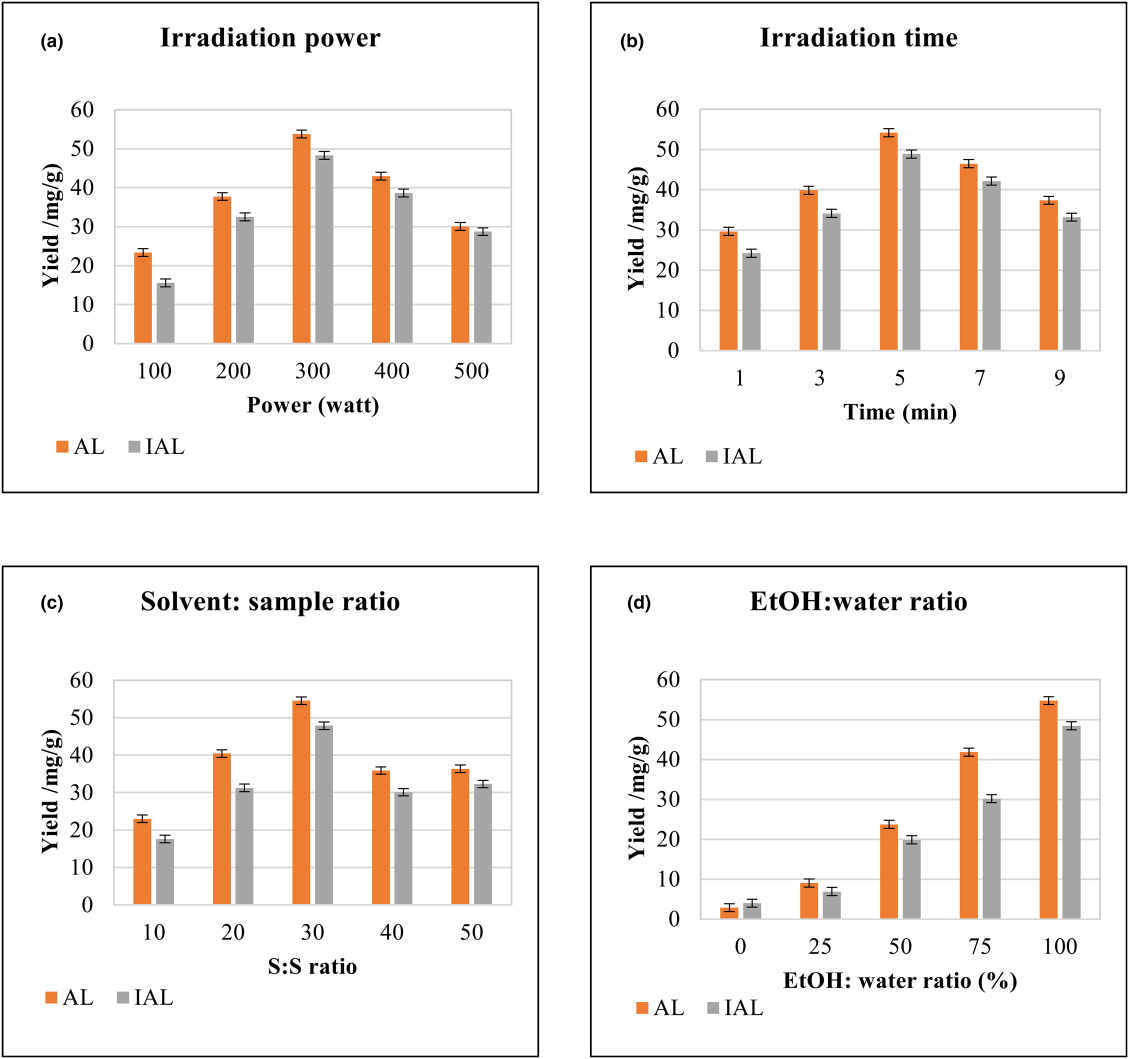
Single‐factor experiments: effects of the power (a), time (b), liquid: sample (c), and ethanol: water ratios (d) of MAE on the yields of AL and IAL of IHRE.

The determination of sesquiterpene lactones using liquid chromatography–tandem mass spectrometry (LC–MS/MS) was realized on a Zivak Tandem Gold (Istanbul, Turkey), equipped with a Zorbax SB‐C18 column (3.0 × 100 mm I.D., particle size 3.5 μm; Agilent). The chromatographic conditions of the LC–MS/MS analysis are summarized in Table S1. The peaks of AL and IAL were defined by comparison with the retention times of the analytical standards, as indicated in Figure 2.

### 2.5 Scanning electron microscope

The microscopic observation on the morphological changes of the *I. helenium* roots after extraction was made through the scanning electron microscope (SEM Quanta 250, FEI Company) with magnifications of ×250, ×500, ×1000, and ×2500. The analysis of samples was conducted at different acceleration voltages between 10 and 20 kV (Ying et al., 2011).

### 2.6 Antibacterial activity

By using the disk diffusion method, the qualitative antibacterial activities of MAE–IHRE and maceration–IHRE were tested against strains of *Staphylococcus aureus*, *Escherichia coli*, and *Lysteria monocytogenes*. Petri dishes containing tryptic soy agar were seeded with diluted 10^2^ CFU/mL concentrations of *S. aureus*, *E. coli*, and *L. monocytogenes*. A sterile filter paper disk (6.0 mm in diameter) was adsorbed with 5, 15, 25, and 50 μL of each extract and evaporated for approximately 10 min at room temperature. At 37°C, the plates were incubated for 24 h. Around the filter disks, antibacterial extracts produced distinct, transparent, and circular zones of inhibition. This positive activity was calculated by measuring the growth inhibition zone (mm) surrounding the disks after 24 h (Corona et al., 2017). A disk loaded with only ethanol was prepared as a negative control.

### 2.7 Antifungal activity

In this study, *Aspergillus niger*, *Penicillium*, and *Fusarium* strains were inoculated onto malt extract agar (MEA) plates from stock culture tubes and incubated at 28°C for 5 days in the dark until sporulation. Mold spores produced by MEA were extracted using a loop, suspended in sterile physiological water, and then adjusted to a concentration of 10^6^ CFU/mL. The agar was inoculated with the malt extract, which was made from this saline and contained roughly 10^6^ CFU/mL of mold spores. Sterile filter paper disks were then placed on top. A sterile filter paper disk (6 mm in diameter) was then impregnated with 5, 15, 25, and 50 μL of each extract and allowed to dry at room temperature for about 10 min. At 28°C, the plates were incubated for 24–48 h. Zones of inhibition (mm) around the disks were measured (Ünal et al., 2012).

### 2.8 Calculation of energy consumption and CO_2_ emissions

The energy consumption was calculated using the following formula: Equation (2) (Gałuszka et al., 2012).

(2) Energy consumption=P×t

where *P* represents the power (W) of the extraction equipment and *t* indicates the extraction time (min).

The water bath and microwave digestion system used in the present study had a power of 1600 and 300 W, respectively. The powers mentioned herein are used as a standard to calculate the energy consumption of conventional and microwave‐assisted extractions of sesquiterpene lactones from the IHRE.

Analysis of energy consumption is a prerequisite for a technology that has the potential to be scaled up to the industrial level. The total energy consumption was calculated according to the electricity consumption of each technology. Carbon emissions were calculated considering that 1 kWh produces 0.8 kg of CO_2_ (Li et al., 2013).

### 2.9 Statistical analysis

All experiments were performed in triplicate, and the results were calculated as mean ± standard deviation. Statistical analysis was conducted using SPSS Statistics Software (IBM version 23, USA). For the RSM, all results obtained from the MAE were studied statistically using the Design Expert software (version 7.0.0, Stat‐Ease Inc.). With this software, analysis of variance (ANOVA) was also performed to determine the plots of the response surfaces and the model building.

## 3 RESULTS AND DISCUSSION

### 3.1 Microwave‐assisted extraction

#### 3.1.1 Effects of power, time, solvent/sample, and ethanol/water ratios

**FIGURE 3:**
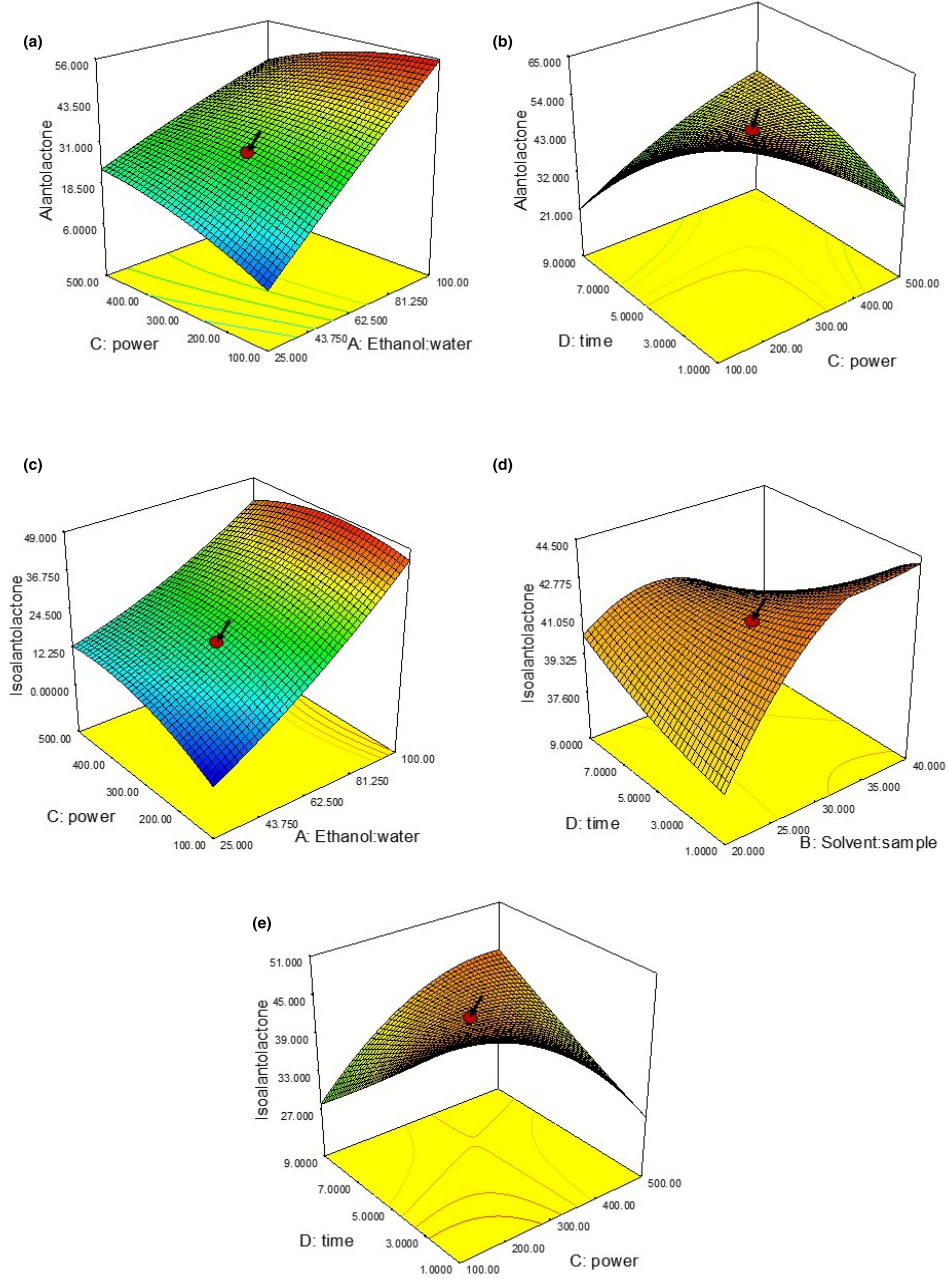
Response surface 3D graphs indicating the significant interactive effects of independent variables on AL and IAL content (a) ethanol/water ratio versus microwave power (AL); (b) microwave time versus microwave power (AL); (c) ethanol/water ratio versus microwave power (IAL); (d) solution/sample ratio versus microwave time (IAL); (e) microwave time versus microwave power (IAL).

As important influencing factors on the yield, the effects of MAE with different power levels, times, L:S ratios, and ethanol/water ratios on the yields of AL and IAL were investigated in the experiment. As represented in Figure 3a, the yields of AL and IAL increased with increasing MAE power between 100 and 300 W and decreased significantly above 300 W. This is thought to be related to the fact that the increase in microwave energy may cause the degradation of sesquiterpene lactones by increasing the temperature in the reaction system. Therefore, the optimum extraction power was determined to be 300 W. At this value, the yields of AL and IAL were found to be 53.77 ± 0.13 and 48.32 ± 0.14 mg/g, respectively. The yields of AL and IAL increased rapidly up to 5 min, decreased between 5 and 7 min, and were then almost unchanged. Therefore, the optimum extraction time was found to be 5 min, with AL and IAL yields of 54.17 ± 0.24 and 48.87 ± 0.22 mg/g, respectively. This is thought to be related to the possibility that longer extraction times may have adverse effects such as degradation or transformation of sesquiterpene lactones. As shown in Figure 3a, the increase in the ethanol/water ratio increased the AL and IAL yields. This was because AL and IAL had good solubility in ethanol and water, as a polar solvent with a high dielectric constant, which could cause their degradation by absorbing more microwave energy and increasing the temperature in the reaction system (Zhao et al., 2015). Thus, 100% was chosen as the optimum ethanol/water ratio. At this value, the yields of AL and IAL were 54.75 ± 0.19 and 48.45 ± 0.17 mg/g, respectively. However, although the L:S ratio increased between 10:1 and 30:1, it decreased after this value and then remained almost unchanged. Therefore, 30:1 was selected as the optimum L:S ratio (AL, 54.53 ± 0.198 mg/g; IAL, 47.87 ± 0.06 mg/g).

#### 3.1.2 Model fitting

The ANOVA results for responses such as those of the AL and IAL values at a 95% confidence level are displayed in Table S2.

Based on the ANOVA data, the fitted FC‐CCD models were applied to show significant regression with a nonsignificant lack of fit and desirable regression coefficients for both responses. The *R* ^2^ values for AL and IAL were observed to be 0.96 and 0.98, respectively, and were systematically close to the unit (1). Therefore, their fit was good, and both quadratic models were workable. The full second‐order polynomial equations for both responses (AL and IAL) describing the AL and IAL contents as a function of the coded factors gained from the experimental design are expressed below (Equations 3 and 4):

(3) $$\begin{array}{cc} \mathrm{AL} & =29.942+11.712^{*}X_{1}+2.1906^{*}X_{2}+1.8959^{*}X_{3}+0.52758^{*}X_{4}-1.6214^{*}{X_{1}}^{*}X_{2}\\ & -2.3646^{*}{X_{1}}^{*}X_{3}-1.7499^{*}{X_{1}}^{*}X_{4}+0.26780^{*}{X_{2}}^{*}X_{3}-1.4405^{*}{X_{2}}^{*}X_{4}\\ & +3.7660^{*}{X_{3}}^{*}X_{4}-0.37083^{*}{X_{1}}^{2}-3.8198^{*}{X_{2}}^{2}-0.66422^{*}{X_{3}}^{2}-0.81193^{*}{X_{4}}^{2} \end{array}$$

(4) $$\begin{array}{cc} \mathrm{IAL} & =20.598+11.364^{*}X_{1}+1.8091^{*}X_{2}+1.2135^{*}X_{3}+0.95199^{*}X_{4}-0.46600^{*}{X_{1}}^{*}X_{2}\\ & -1.3809^{*}{X_{1}}^{*}X_{3}-0.87884^{*}{X_{1}}^{*}X_{4}+0.32147^{*}{X_{2}}^{*}X_{3}-1.1130^{*}{X_{2}}^{*}X_{4}\\ & +2.3290^{*}{X_{3}}^{*}X_{4}-1.3028^{*}{X_{1}}^{2}-2.3743^{*}{X_{2}}^{2}-1.1206^{*}{X_{3}}^{2}-0.087634^{*}{X_{4}}^{2} \end{array}$$

**FIGURE 4:**
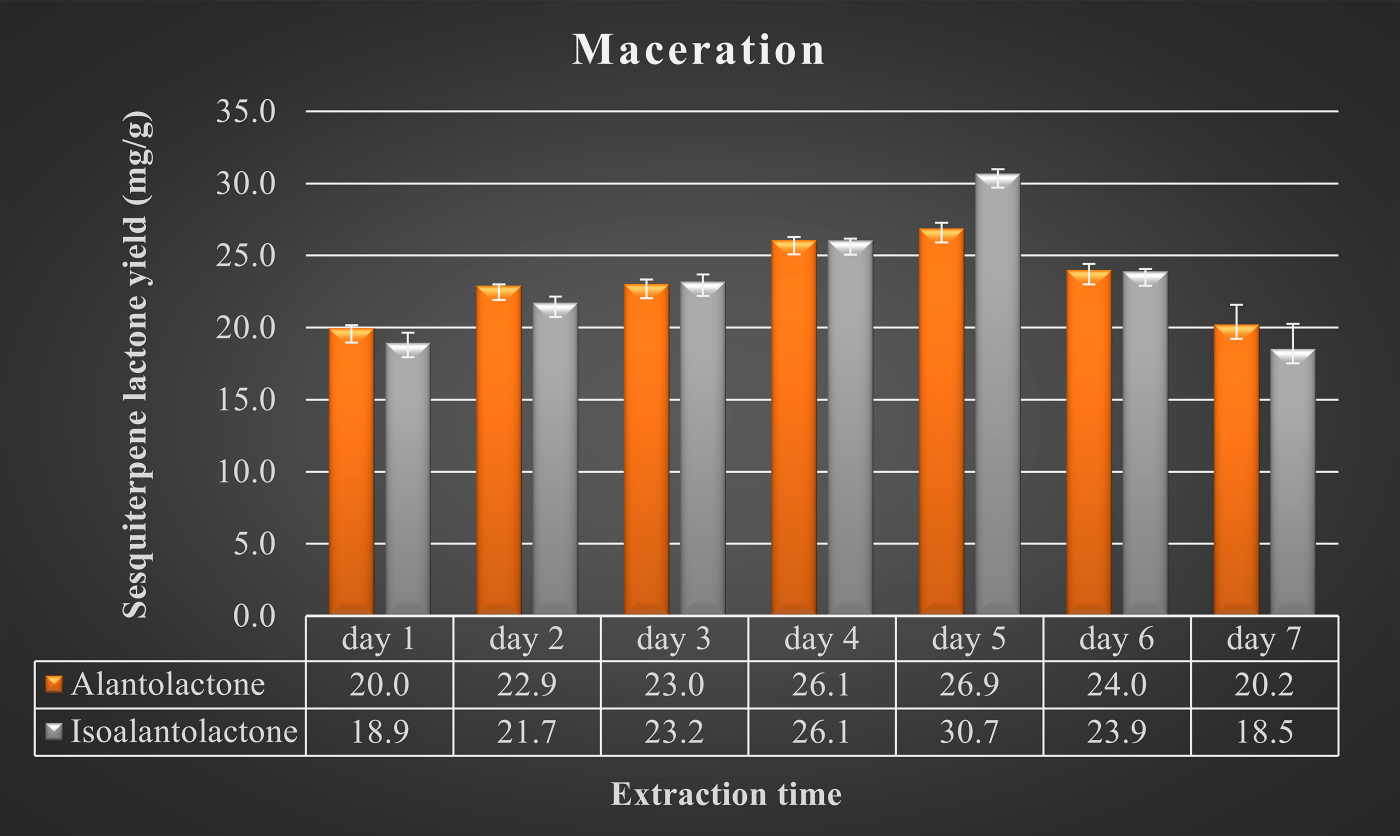
Single‐factor experiments: time (day) parameter of maceration on the yields of AL and IAL of IHRE.

A three‐dimensional graphical representation of both responses of the experimental design is presented in Figure 4 as a simultaneous function of two independent variables according to their significance to each response.

The predicted *R* ^2^ of 0.78785 for the AL and 0.93639 for the IAL were in reasonable agreement with the adjusted *R* ^2^ of 0.91370 for the AL and 0.96724 for the IAL. Likewise, the range of the predicted AL and IAL yields was in close accord with the average prediction error. However, higher adequacy precision values of 18.132 and 32.232 for the AL and IAL, respectively, were obtained, providing a good indicator for the signal‐to‐noise ratio (S/N >4) and indicating that both quadratic models could navigate the design space.

As a result, in the range of the experimental factors selected in the present study, the quadratic models for the MAE technique were appropriate and sufficient for forecasting the AL and IAL yields.

#### 3.1.3 Effect of process variables on the extraction yield of sesquiterpene lactones

As shown in Figure 4, the interaction effects of the ethanol/water ratio and power of exposure (*X*_1_ *X*_3_) and power of exposure and time of exposure (*X*_3_ *X*_4_) were synergistic on the AL yield, whereas the ethanol/water ratio and power of exposure (*X*_1_ *X*_3_), L:S ratio and time of exposure (*X*_2_ *X*_4_), and power of exposure and time of exposure (*X*_3_ *X*_4_) were synergistic on the IAL yield. However, the interaction effect of the other factors was not significant. Considering the significant quadratic effects of all factors according to the ANOVA data (Table S2), this suggests that at higher levels, these factors may have a negative impact on AL and IAL yields (Table 1).

At the designated points of experimental runs constructed by Design Expert, AL, and IAL yields ranged from 2.87 to 54.99 mg/g and 1.82 to 48.40 mg/g, respectively. Most importantly, the highest AL and IAL yields (54.99 ± 0.107 and 48.40 ± 0.191 mg/g, respectively) were reported at an MAE exposure time of 5 min, an exposure power of 300 W, an L:S ratio of 30:1 (mL/mg), and an ethanol/water ratio of 100:0 (Table 1).

According to the desirability function method, the optimum extraction conditions were an ethanol/water ratio of 99.95%, an L:S ratio of 44.57 mL/mg, an exposure power of 212.8 W, and an exposure time of 7.00 min for microwave extraction. The desirability value of the optimum parameters of MAE was 100%. Under these conditions, the predicted values for AL and IAL were 56.89 ± 0.23 and 48.77 ± 0.02 mg/g, respectively, whereas the recovered values were 55.27 ± 1.11 and 47.97 ± 0.82 mg/g, respectively.

To the best of our knowledge, there has only been one study on the optimization of AL and IAL extraction using the MAE technique. In this study, only a single‐factor experiment was performed, and the interaction effect of all factors was not observed. In addition, the effect of microwave exposure power on AL and IAL extraction yields was not investigated. Zhao et al. (2015) found the yield of AL and IAL to be 31.83 and 21.25 mg/g, respectively, in their study, whereas we found it to be 54.99 ± 0.11 and 48.40 ± 0.19 mg/g, respectively. This shows that we achieved nearly two times the yield for AL and more than two times the yield for IAL compared with Zhao et al. (2015) using our optimization design.

In addition, Petkova et al. (2017) used MAE for the extraction of carbohydrates and phenolic compounds (total phenol, flavonoids, and phenolic acids) in *I. helenium*, but the extraction of sesquiterpene lactones was not performed using this technique.

### 3.2 Maceration

The effect of maceration at different times on the yield of AL and IAL was investigated in the experiment. As represented in Figure 3b, the yield of AL and IAL increased with increasing time between 1 and 4–5 days and decreased significantly from 5 to 7 days. The most significant changes in the yield of AL and IAL with increasing time were concentrated in the region of 3–6 days. In addition, the maximum release of AL (26.89 ± 0.4 mg/g) and IAL (30.69 ± 0.3 mg/g) was observed after 4 days (Figure 3b). Briefly, the results indicate that the AL and IAL yields did not increase continuously with increasing time.

### 3.3 Morphological analysis

**FIGURE 5:**
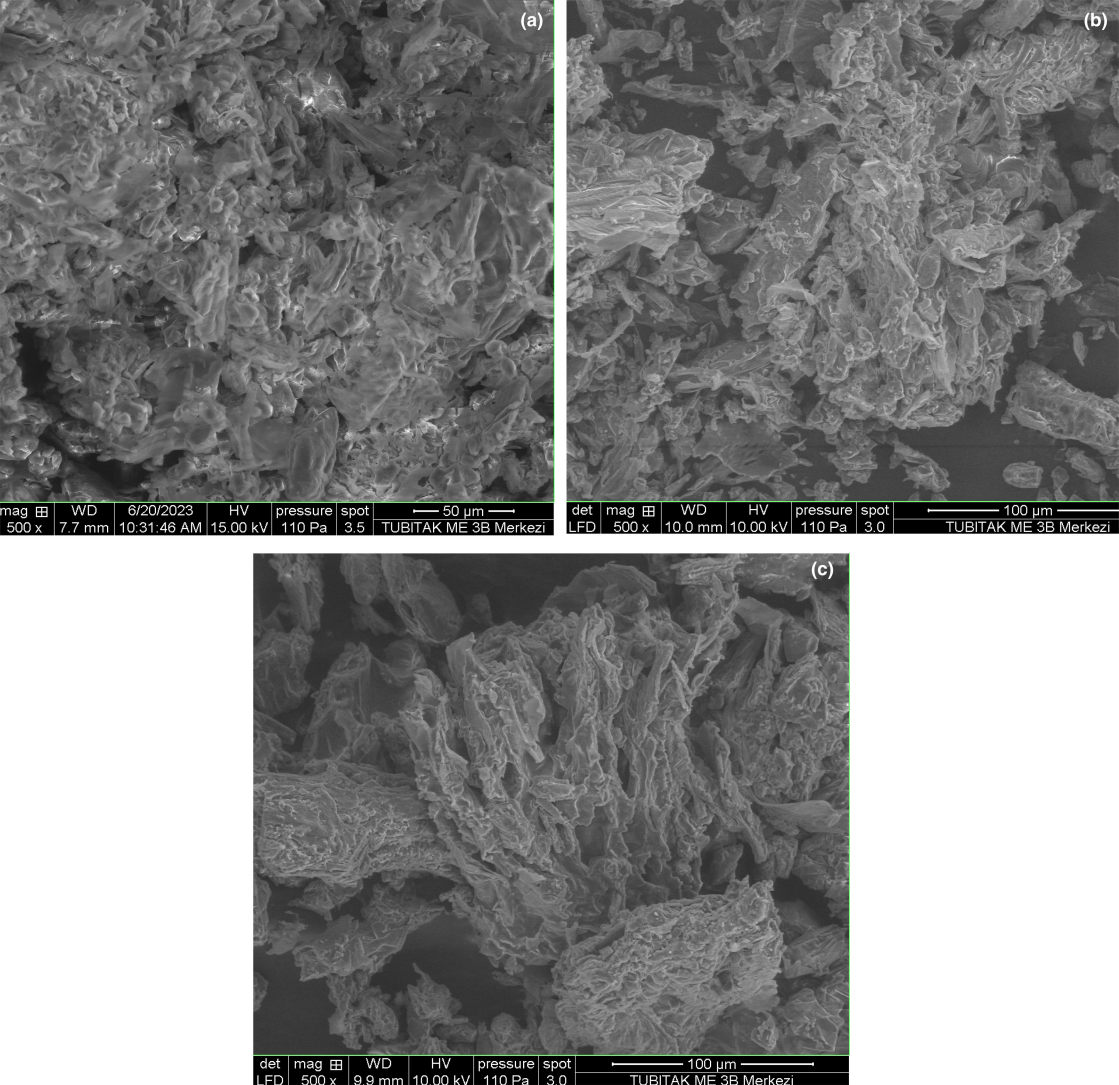
SEM results of *I. helenium* root (a) untreated (control) and treated by (b) maceration and (c) MAE.

Physical changes in the plant material are related to extraction efficiency (Ying et al., 2011). The morphological changes of MAE and conventional extraction techniques were characterized by SEM, and the results are demonstrated in Figure 5. The surface morphology of *I. helenium* root, untreated (control) and treated by MAE and maceration, was observed at different magnification levels.

Different extraction processes resulted in noticeable physical changes (Figure 5). Compared with the surface of untreated *I. helenium* roots displayed in Figure 5a, the roots with maceration displayed slight damage on the sample surface, as illustrated in Figure 5b. On the other hand, those treated by MAE (Figure 5c) displayed obvious cell disruption. This result was also in agreement with Ying et al. (2011). The solvent in the maceration gradually dispersed along the cell walls of the tissues and dissolved and removed the targeted compounds. Therefore, slight damage occurred in the structure of the tissues. However, because high pressure was created inside the plant materials in MAE, the physical characteristics of the cell walls of the tissues were considerably altered, and the cell structure of the tissues was disturbed (Ying et al., 2011). According to the morphological changes in *I. helenium* roots before and after the extraction procedure, SEM data confirmed that MAE is a significantly more efficient extraction technique than maceration.

### 3.4 Antimicrobial activity

**FIGURE 6:**
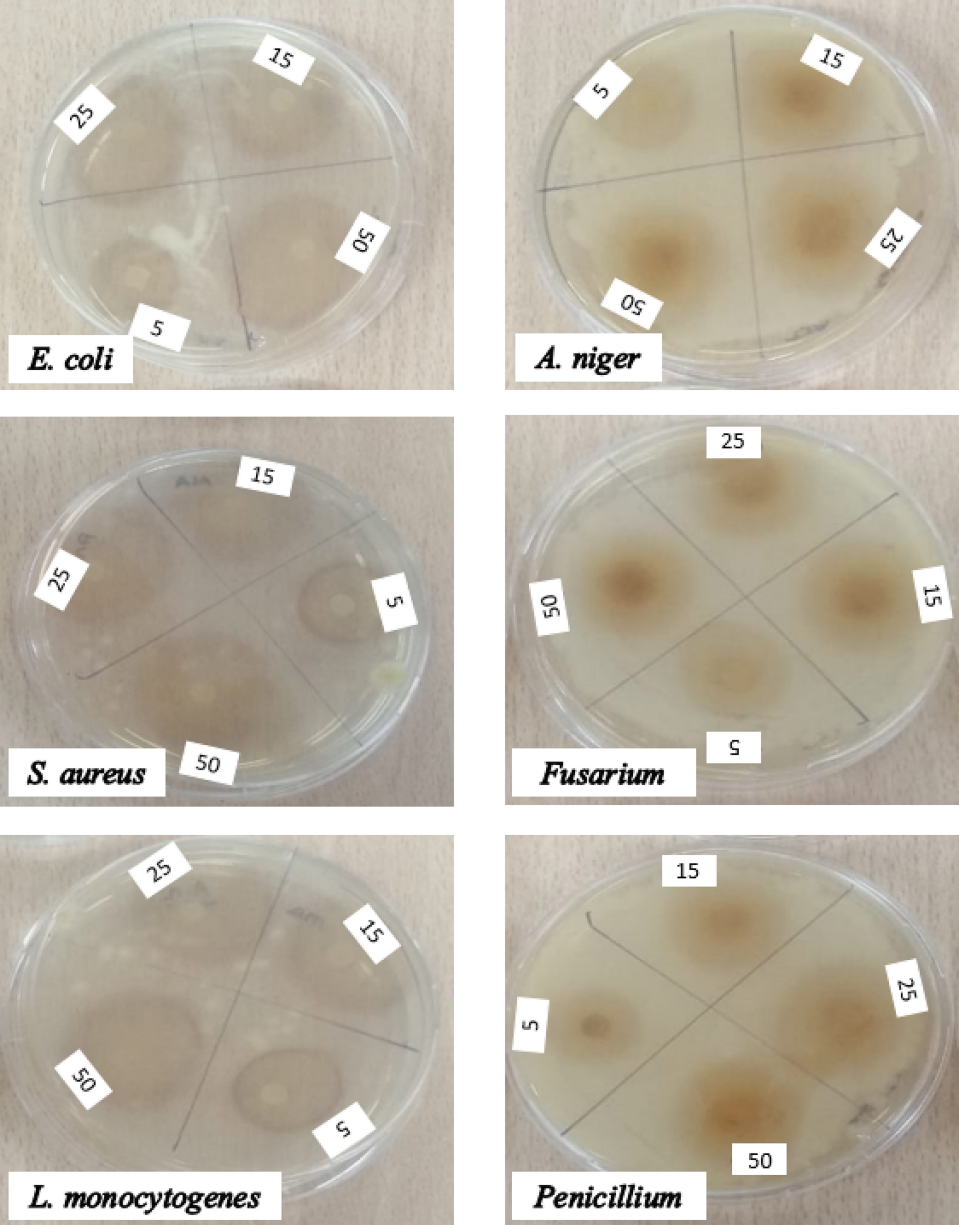
Agar disk diffusion assay showing the antibacterial and antifungal effects of MAE–IHRE.

Some studies have shown that plant extracts have antibacterial and antifungal activities, which may be related to the presence of bioactive compounds (Gökbulut et al., 2013), (Flores et al., 2023). Therefore, in this study, the disk diffusion test was designed to determine the potential of MAE–IHRE and maceration–IHRE to inhibit the growth of some strains. Within this scope, the changes in the diameters of the inhibition zone of the IHRE in the best extraction conditions (5 min, 30:1 (L:S ratio, mL/mg), 100% ethanol, 300 W power for MAE; 5 days, 30:1 (L:S ratio, mL/mg), 100% ethanol for maceration) against Gram‐positive and Gram‐negative strains, fungi, and positive controls were investigated. The values are presented in Table 2. According to the experiments (Table 2), the inhibitory zones changed in a concentration‐dependent manner and were observed at all concentrations in all organisms tested. Furthermore, MAE–IHRE showed remarkable antimicrobial activity against all these pathogens compared to the antibacterial activity of Chloramphenicol (25 mcg) and the antifungal activity of Fluconazole (25 mcg). *E. coli* (21.5 ± 0.71 mm, 50 μL concentration), with the smallest inhibition zones, was the least sensitive test organism for the maceration technique, whereas *A. niger* (29.25 ± 1.06 mm) and *Penicillium* (29.5 ± 0.71 mm), with the largest inhibition zones, were the most sensitive organisms (Figure 6). However, for MAE, *L. monocytegenes* with the smallest inhibition zone (27.5 ± 0.71 mm), was the least sensitive test organism, and *A. niger* (34.75 ± 1.06 mm) and *Penicillium* (34.5 ± 0.71 mm), with the largest inhibition zones, were the most sensitive organisms (Figure 6). Consequently, all data on the zones of inhibition revealed that MAE–IHRE was more effective than maceration–IHRE against all organisms tested in this study. Kenny et al. (2022) identified AL, IAL, and igalan as the natural products contributing to the antimicrobial activity observed in vitro. This is thought to be due to the fact that MAE–IHRE contains higher AL and IAL than maceration–IHRE.

**TABLE 2:** Antibacterial and antifungal activities of *Inula helenium* root extracts.

		Zone of inhibition (mm)					
		Concentration (μL)					
		5	15	25	50	CAP (25 μg)	Fluconazole (25 μg)
*E. coli*	MAE	5.75 ± 0.35^A^	15.75 ± 0.35^A^	20.50 ± 0.71^A^	29.5 ± 0.71^A^	17.5 ± 0.35	–
	Maceration	3.75 ± 0.35^B^	9.50 ± 0.71^B^	14.75 ± 0.35^B^	21.5 ± 0.71^B^		
*S. aureus*	MAE	5.5 ± 0.71^A^	14.5 ± 0.71^A^	18 ± 0.00^A^	29.25 ± 0.35^A^	17.75 ± 0.71	–
	Maceration	5.25 ± 0.35^A^	10.25 ± 0.35^B^	15 ± 0.00^B^	24.75 ± 0.35^B^		
*L. monocytegenes*	MAE	6.25 ± 0.71^A^	18.5 ± 0.71^A^	25 ± 0.00^A^	27.5 ± 0.71^A^	21.00 ± 0.35	–
	Maceration	5.75 ± 0.35^A^	15.5 ± 0.71^B^	22 ± 0.00^B^	25.5 ± 0.71^B^		
*A. niger*	MAE	10.5 ± 0.71^A^	25.5 ± 0.71^A^	29.5 ± 0.71^A^	34.75 ± 1.06^A^	–	20.5 ± 0.71
	Maceration	7 ± 0.00^B^	20.5 ± 0.71^B^	25.5 ± 0.71^B^	29.25 ± 1.06^B^		
*Fusarium* spp	MAE	7 ± 0.00^A^	19.5 ± 0.71^A^	26.75 ± 0.35^A^	30.5 ± 0.71^A^	–	13.25 ± 1.06
	Maceration	4.25 ± 0.35^B^	12.5 ± 0.71^B^	18 ± 0.00^B^	23.75 ± 0.35^B^		
*Penicillium* spp	MAE	10 ± 0.00^A^	25.75 ± 1.06^A^	29.5 ± 0.71^A^	34.5 ± 0.71^A^	–	19.75 ± 1.06
	Maceration	7.5 ± 0.71^B^	20 ± 0.00^B^	24.25 ± 0.35^B^	29.5 ± 0.71^B^		

*Note*: Values are mean ± SE. Different superscript uppercase letters on the same column mean significant differences in the inhibition diameter produced by extracts of *I. helenium*; A to B: highest to lowest. CAP, chloramphenicol; MAE, microwave‐assisted extraction; Gram‐positive bacteria: *S. aureus*, *L. monocytegenes*; Gram‐negative bacteria: *E. coli*.

Buza et al. (2022) applied traditional extraction (maceration) to *I. helenium* roots with 70% ethanol for 10 days and performed antibacterial activity analysis on this extract. The obtained values for the inhibition zone diameters in *I. helenium* root extracts were found to be 10.67 ± 0.47 and 16.33 ± 0.47 mm for *E. coli* and *S. aureus*, respectively. In our study, these values were also found to be 28.5 ± 0.71 and 129.25 ± 0.35 mm for *E. coli* and *S. aureus* in MAE–IHRE, respectively. Accordingly, it is thought that the reason for the higher antibacterial activity in our study is the higher concentration of ethanol used as a solvent, the shorter maceration time (best efficiency on day 5 for AL and IAL), and the more effective extraction of bioactive compounds using the MAE technique.

### 3.5 Energy consumption and CO_2_ emissions

For industrial viability, energy requirements, and carbon emissions are important parameters to evaluate. Therefore, energy consumption and carbon emissions values were calculated in the current study. As expected, the optimization of MAE process parameters for the extraction of sesquiterpene lactones using RSM–FC‐CCD resulted in a reduction in MAE exposure time and thus a reduction in energy consumption and carbon emissions. As depicted in Table 3, the energy consumption and carbon emissions were 96 kWh and 76.8 kg CO_2_/kg extract for maceration and 0.42 kWh and 0.34 kg CO_2_/kg extract for MAE, respectively, for recovering sesquiterpene lactones from IHRE. Accordingly, the optimized process, MAE, reduced the energy input and carbon emissions 228.6‐fold and the exposure time 43.4‐fold when compared with conventional extraction (maceration).

**TABLE 3:** Comparison of techniques in terms of AL and IAL efficiencies, energy consumption, and carbon emissions.

	Maceration	MAE
AL yield (mg/g)	26.89 ± 0.4^B^	54.99 ± 0.11^A^
IAL yield (mg/g)	30.69 ± 0.3^B^	48.40 ± 0.19^A^
Energy consumption (kWh/kg extract)	96	0.42
CO_2_ emission (kg CO_2_/kg extract)	76.8	0.34

*Note*: Values are mean ± SE. Different letters mean the statistical difference between groups.

Regarding the current findings, Mary Leema et al. (2022) reported a 15‐fold reduction in exposure time and a 5.35‐fold reduction in energy consumption for the MAE of lutein from *Chlorella sorokiniana*, whereas Vila Verde et al. (2018) reported a 14‐fold and 6‐fold reduction in exposure time and energy consumption, respectively, for the MAE of terpenes from *Pterodon emarginatus*. Additionally, Périno‐Issartier et al. (2011) declared that MAE reduces extraction time (15‐fold) and energy consumption (6‐fold) compared with conventional extraction. The energy efficiency of MAE is due to the synergistic effect of heat and mass transfer in the same direction (Périno‐Issartier et al., 2011).

Sarkar et al. (2021) applied MAE with an energy consumption of 0.025 kWh and 20 kg CO_2_/kg extract and maceration with an energy consumption of 0.05 kWh and 40 kg CO_2_/kg extract for production of inulin from *Pachyrhizus erosus* root. They reported a 50% reduction in carbon footprint and energy consumption for MAE over the conventional method. Similar observation was reported by Vinatoru et al. (2017) and Jacotet‐Navarro et al. (2015). In summary, as a green extraction technique, MAE promotes increased functional and economic applications of sesquiterpene lactones by significantly reducing processing times, energy costs, and carbon emissions while increasing yields (Table 3).

## 4 CONCLUSIONS

The optimized conditions for the MAE efficiency of sesquiterpene lactones were found at a duration of 5 min, an ethanol/water ratio of 100:0, a power of 300 W, and an L:S ratio of 30:1 (mL/g). Under these conditions, the highest extraction yields were predicted at 56.89 ± 0.23 and 48.77 ± 0.02 mg/g for AL and IAL, respectively. These outcomes are consistent with results from other validation experiments (55.27 ± 1.11 mg/g for AL and 47.97 ± 0.82 mg/g for IAL). Accordingly, the MAE enabled higher AL and IAL yields compared to maceration. These results were also confirmed by SEM images. Moreover, our findings displayed higher antibacterial and antifungal activities for MAE–IHRE than for maceration–IHRE. Consequently, MAE was demonstrated to be a sustainable alternative to conventional extraction, showing the potential to increase extraction yield and decrease extraction time and energy consumption. Furthermore, in terms of reducing carbon emissions, the use of the MAE technique was revealed to be a much more accurate option. Therefore, the MAE technique should be considered feasible for mass production at the industrial level.

## AUTHOR CONTRIBUTIONS

**Fahriye Şeyma Özcan:** Conceptualization (equal); data curation (equal); investigation (equal); methodology (equal); project administration (equal); writing – original draft (lead). **Hilal Dikmen:** Data curation (equal); formal analysis (equal). **Nihat Özcan:** Conceptualization (equal); data curation (equal); investigation (equal); methodology (equal); validation (equal); writing – review and editing (equal). **Özlem Çetİn:** Resources (equal). **Mustafa Çelİk:** Resources (equal). **Antoaneta Trendafilova:** Data curation (equal); project administration (equal).

## FUNDING INFORMATION

This study was financially supported by the Scientific and Technological Research Council of Turkey “TUBITAK” Contract No.121N195 and Bulgarian Academy of Sciences “BAS” Contract No. IC‐TR/6/2022–2024.

## CONFLICT OF INTEREST STATEMENT

The authors have no conflicts of interest to declare.

## Supporting information


Table S1

## Data Availability

The data that support the findings of this study are available from the corresponding author upon reasonable request.
