# Supplementary material for: Microwave‐assisted extraction optimization of sesquiterpene lactones from Inula helenium roots: A sustainable approach to reduce energy consumption and carbon footprint
Source: Food Sci Nutr. 2023 Oct 13;12(1):255–67. doi: 10.1002/fsn3.3775 (PMC10804079; doi:10.1002/fsn3.3775)
Supplement: Supplementary file 1 — Table S1 [file FSN3-12-255-s001.docx]

**SUPPLEMENTARY** **MATERIALS**

**Table 1** Chromatographic conditions of LC–MS/MS analysis.

| Parameters | Chromatographic conditions | |
| --- | --- | --- |
| Mobil phase A | Deionised water and 0.1 % formic acid | |
| Mobil phase B | MeOH: ACN (90:10) | |
| Pump programme | 00:00–04:00 min, 10% B | |
|  | 04:00–27:00 min, 55% B | |
|  | 27:00–36:00 min, 80% B | |
|  | 36:00–38:00 min, 10% B | |
| Ion spray voltage | 5000 V | |
| Nebuliser gas (nitrogen) | 35 psi | |
| Source temperature | 50 °C | |
| Drying gas temperature | 350 °C | |
| Analytes | Alantolactone | Isoalantolactone |
| Precursor ion | 233.15 | 233.15 |
| Daughter ion | 105.06 | 105.07 |
| Capillary voltage | 30 | 30 |
| Collision energy | 25 | 25 |
| Retention time | 30.11 | 29.79 |

**Table 2** Analysis of variance of the MAE of sesquiterpene lactones.

|  |  | AL | | IAL | |  |
| --- | --- | --- | --- | --- | --- | --- |
| Source | df | SS | p^a^ | SS | p^a^ | Significance |
| Model | 14 | 4346.0 | **< 0.0001** | 3650.7 | **< 0.0001** | **Significant** |
| A | 1 | 3292.4 | **< 0.0001** | 3099.5 | **< 0.0001** |  |
| B | 1 | 115.17 | **0.0106** | 78.547 | **0.0006** |  |
| C | 1 | 86.262 | **0.0234** | 35.341 | **0.0109** |  |
| D | 1 | 6.6802 | 0.4932 | 21.751 | **0.0379** |  |
| AB | 1 | 42.065 | 0.0983 | 3.4744 | 0.3772 |  |
| AC | 1 | 89.461 | **0.0213** | 30.510 | **0.0166** |  |
| AD | 1 | 48.997 | 0.0765 | 12.358 | 0.1067 |  |
| BC | 1 | 1.1475 | 0.7749 | 1.6535 | 0.5396 |  |
| BD | 1 | 33.199 | 0.1382 | 19.821 | **0.0462** |  |
| CD | 1 | 226.92 | **0.0096** | 86.785 | **0.0004** |  |
| A^2^ | 1 | 3.7719 | 0.6053 | 46.552 | **0.0046** |  |
| B^2^ | 1 | 400.20 | **< 0.0001** | 154.62 | **< 0.0001** |  |
| C^2^ | 1 | 12.101 | 0.3594 | 34.441 | **0.0118** |  |
| D^2^ | 1 | 18.082 | 0.2659 | 0.21064 | 0.8257 |  |
| Lack of Fit | 10 | 155.71 | 0.3038 | 33.701 | 0.5765 | **Not significant** |
| R^2^ |  |  | 0.9554 |  | 0.9831 |  |
| Adjusted R^2^ |  |  | 0.9137 |  | 0.9672 |  |
| CV (%) |  |  | 14.481 |  | 10.829 |  |

df: degree of freedom.

SS: sum of squares.

CV: coefficient of variation.

A: linear effect of ethanol (EtOH)/water.

B: linear effect of solvent/solid (S:S).

C: linear effect of power (watt).

D: linear effect of time (min).

A^2^: quadratic effect of EtOH/water.

B^2^: quadratic effect of S:S.

C^2^: quadratic effect of power.

D^2^: quadratic effect of time.

AB: interaction effect of EtOH/water (S:S).

AC: interaction effect of EtOH/water (power).

AD: interaction effect of EtOH/water (time).

BC: interaction effect of S:S (power).

CD: interaction effect of power (time).

^a^ Bold values indicate statistical significance (p ≤ 0.05).
